# Editorial: Advanced electrode materials: towards high-performance battery electrodes via ingenious materials design and fabrication

**DOI:** 10.3389/fchem.2023.1205552

**Published:** 2023-05-10

**Authors:** Guowei Zhou, Xiaojun Zhang, Derrick Fam, Hongda Li, Junhao Liu

**Affiliations:** ^1^ Key Laboratory of Fine Chemicals in Universities of Shandong, Jinan Engineering Laboratory for Multi-Scale Functional Materials, School of Chemistry and Chemical Engineering, Qilu University of Technology (Shandong Academy of Sciences), Jinan, China; ^2^ College of Chemistry and Materials Science, Anhui Normal University, Wuhu, China; ^3^ School of Materials Science and Engineering, Nanyang Technological University, Singapore, Singapore; ^4^ Department of Mechanical Engineering, National University of Singapore, Singapore, Singapore; ^5^ School of Microelectronics and Materials Engineering, Guangxi University of Science and Technology, Liuzhou, China; ^6^ Key Laboratory of Green Process and Engineering, Institute of Process Engineering, Chinese Academy of Sciences, Beijing, China

**Keywords:** lithium-ion battery, supercapacitor, electrochemical reduction of CO_2_, 2D metal tellurides, synergetic effect

Innovative morphology and structural design in advanced electrode materials at the nanoscale scale provide tremendous promise to further the creation of high-performance electrodes. Due to capacity deterioration and insufficient energy densities, which prevent the practical adoption of these technologies in the future. The search for high-performance electrode materials for batteries other than Li-ion batteries remains difficult.

We appreciate all the authors and consider it a pleasure to have hosted this research subject on Advanced Electrode Materials: Towards High-Performance Battery Electrodes through Ingenious Materials Design and Fabrication. This study’s objective is to give a comprehensive summary of current developments in smart nanostructured materials that have a big influence on the batteries operate behavior electrochemically. For high energy density and durable electrode materials, several workable electrode design strategies have been developed, primarily in terms of alloy nanostructure (Zeng et al.), porous materials (Li et al.), well-designed spinel anode (Wang et al.), as well as a review on the recent progress of two-dimensional (2D) metal tellurides electrodes (Guo et al.).

The well-designed nanostructured Cu-Al catalysts, which are made of Cu_2_O modified by Al and prepared by a green microwave-assisted solvothermal process, can efficiently and selectively convert CO_2_ to CO and HCOOH. At 1.0 and 1.3 V (*vs*. RHE), good current densities of 67 and 130 mA·cm^−2^ are attained, respectively. When the electrolyte was changed to KOH, an impressive selectivity for C_2_H_4_ production was observed, as high as 20%, and the current densities reached 146 and 222 mA·cm^−2^ at 1.0 and 1.3 V (*vs*. RHE), respectively. The work suggested a method for producing Cu-Al materials that is economic friendship, ecologically friendly, and energy-efficient, enabling their mass manufacturing.

A type of Co/C material with a stable hollow structure that offers a lot of space for particle shrinkage and expansion was proposed for the electrodes in supercapacitors. The materials displayed outstanding specific capacitance (400 F·g^−1^, 0.5 A·g^−1^) and stability (90%, 10,000 cycles). The electrochemical performance was strongly impacted by the various ratio concentrations in the structure. This research demonstrated that the carbon-based materials’ ability to store energy was dependent on the interaction between their component and mesoporous structures.

The self-templating solvothermal approach was suggested to create rambutan-like Co_0.5_Ni_0.5_Fe_2_O_4_ for employing as an anode in high-performance Li-ion batteries. Co_0.5_Ni_0.5_Fe_2_O_4_, which was made by assembling many nanosheets, has a special chemical structure that makes it possible to efficiently buffer the volume change that happens while charging and discharging. The partial substitution of Ni in NiFe_2_O_4_ results in Co_0.5_Ni_0.5_Fe_2_O_4_, and it is expected that the cohabitation of nickel and cobalt would result in more efficient redox reactions. The rambutan-like Co_0.5_Ni_0.5_Fe_2_O_4_ anode material might have a specific capacity of 963 mA·h·g^−1^ after 200 cycles at a current density of 500 mA·g^−1^. More metal oxide can be produced using this type of synthesis method, which will be used to develop potential Li-ion batteries anodes.

Finally, the exceptional physical and chemical benefits as well as their robust metallic characteristics make two-dimensional metal tellurides potential as electrodes. The fabrication of two-dimensional metal tellurides and their use in electrode materials have recently advanced. First, a summary of the most popular preparation techniques is provided, including aqueous/solvent thermal, chemical vapor deposition, and electrodeposition. Following that, areas like electrocatalysis, lithium-sulfur batteries, lithium/sodium ion anode materials, and metal tellurides in supercapacitors are covered.

More than 450 total downloads and 4,400 total views achieved for this Research Topic. As the guest editors of this issue, we would like to thank all the authors, reviewers, and the editorial staff of Frontiers in Chemistry for the great support.

